# Motilin Comparative Study: Structure, Distribution, Receptors, and Gastrointestinal Motility

**DOI:** 10.3389/fendo.2021.700884

**Published:** 2021-08-23

**Authors:** Takio Kitazawa, Hiroyuki Kaiya

**Affiliations:** ^1^Comparative Animal Pharmacology, Department of Veterinary Science, Rakuno Gakuen University, Ebetsu, Japan; ^2^Department of Biochemistry, National Cerebral and Cardiovascular Center Research Institute, Suita, Japan

**Keywords:** motilin, motilin receptor, gastrointestinal contractility, enteric nerves, smooth muscle, vagus afferent nerves, comparative biology

## Abstract

Motilin, produced in endocrine cells in the mucosa of the upper intestine, is an important regulator of gastrointestinal (GI) motility and mediates the phase III of interdigestive migrating motor complex (MMC) in the stomach of humans, dogs and house musk shrews through the specific motilin receptor (MLN-R). Motilin-induced MMC contributes to the maintenance of normal GI functions and transmits a hunger signal from the stomach to the brain. Motilin has been identified in various mammals, but the physiological roles of motilin in regulating GI motility in these mammals are well not understood due to inconsistencies between studies conducted on different species using a range of experimental conditions. Motilin orthologs have been identified in non-mammalian vertebrates, and the sequence of avian motilin is relatively close to that of mammals, but reptile, amphibian and fish motilins show distinctive different sequences. The MLN-R has also been identified in mammals and non-mammalian vertebrates, and can be divided into two main groups: mammal/bird/reptile/amphibian clade and fish clade. Almost 50 years have passed since discovery of motilin, here we reviewed the structure, distribution, receptor and the GI motility regulatory function of motilin in vertebrates from fish to mammals.

## Introduction

Motilin was identified in the 1970s from the mucosa of the porcine upper intestine as a stimulant of gastric motility (1–3). Brown and colleagues examined the effects of duodenal alkalinization on pressure of the gastric pouch and found that alkalinization caused an increase in pressure of the pouch. Since the pouch was isolated from the autonomic nerves, it was thought that alkalinization induced the release of substances from the duodenal mucosa that stimulate motility. Duodenal extracts of pigs were examined for their gastric motor-stimulating activity, and motilin was separated as a distinct polypeptide. Porcine motilin was found to be a 22-amino acid peptide with a primary sequence of FVPIFTYGEL QRMQEKERNKGQ (1–3). Later, the presence of motilin was shown, and its sequence was determined in rabbits (*Leporinae Trouessart*), humans (*Homo sapiens*), dogs (*Canis lupus familiaris*), cats (*Felis silvestris catus*), cows (*Bos taurus*) and sheep (*Ovis aries*). Although there are some differences in amino acid sequence, the N-terminal sequences are highly conserved among mammals (4–7) (**Table 1**). Curiously, in an early study, motilin did not cause contractions of rat and guinea-pig GI tract (9), and later molecular studies indicated the lack of motilin and/or its receptor in rodents including mice (10–12). Inability of the rodents for motilin study is one of the obstacles for performing extensive physiological studies on the functions of motilin.

**Table 1:** Representative information on motilin in various vertebrates.

Scientific name	Common name	NCBI Transcript #	NCBI Protein #	Mature motilin sequence
**Mammals**				
Homo sapiens	Human	NM_002418	NP_002409	**FVPIFTYGELQRMQ--EKERNK-GQ**
Bos taurus	Cattle	XM_010818020.3	XP_010816322	FVPIFTYGEVQRMQ--EKERYK-GQ
Canis lupus familiaris	Dog	XM_022425739	XP_022281447	FVPIFTHSELQKIR--EKERNK-GQ
Cavia porcellus	Domestic guinea pig	NM_001172860.2	NP_001166331.2	FIPIFTYSELRRTQ--EREQNK-GL
Sorex araneus	European shrew	XM_004617716	XP_004617773	FVPIFTHSELQRMQ--EKEQNK-GR
Monodelphis domestica	Gray short-tailed opossum	XM_007483690.2	XP_007483752	FVPIFTYSDVQRMQ--EKERNK-GQ
Equus caballus	Horse	XM_023624006	XP_023479774	FVPIFTYSELQRMQ--EKERNR-GQ
Sus scrofa	Pig	NM_214235	NP_999400	FVPSFTYGELQRMQ--EKERNK-GQ
Oryctolagus cuniculus	Rabbit	NM_001101699	NP_001095169	FVPIFTYSELQRMQ--ERERNR-GH
Suncus murinus	House musks shrew	AB325968	BAI66099	FMPIFTYGELQKMQ--EKEQNK-GQ
Felis catus	Domestic cat	NM_001009278	NP_001009278	FVPIFTHSELQRIR--EKERNK-GQ
Macaca mulatta	Rhesus monkey	NM_001032807	NP_001027979	FVPIFTYGELQRMQ--EKERSK-GQ
Ovis aries	Sheep	NM_001009439	NP_001009439	FVPIFTYGEVQRMQ--EKERYK-GQ
**Birds**				
Lonchura striata domestica	Bengalese finch	XM_031506971	XP_031362831	FMPFFTQSDFQKMQ--EKERNKAGQ
Gallus gallus	Chicken	NM_001305129	NP_001292058	FVPFFTQSDIQKMQ--EKERNK-GQ
Aquila chrysaetos chrysaetos	Golden eagle	XM_030000337	XP_029856197	FVPFFTKSDFQKMQ--EKERNKGGQ
Coturnix japonica	Japanese quail	XM_015885100.2	XP_015740586	FVPFFTQSDFQKMQ--EKERNK-GQ
Apteryx rowi	Okarito brown kiwi	XM_026056740	XP_025912525	FLPFFTQSDFRKMQ--EKERNK-GQ
Phasianus colchicus	Ring-necked pheasant	XM_031605951	XP_031461811	FVPFFTQSDIQKMQ--EKERIK-GQ
Columba livia	Rock pigeon	XM_021281156	XP_021136831	FVPFFTQSDRFKMQLQEKERNKAGQ
Meleagris gallopavo	Turkey	XM_010724334.3	XP_010722636	FVPFFTQSDIQKMQ--EKERIK-GQ
**Reptiles**				
Alligator mississippiensis	American alligator	XM_019484898	XP_019340443	FLPIFTHSDMQRMQ--ERERNK-GQ
Crocodylus porosus	Australian saltwater crocodile	XM_019546714	XP_019402259	FLPIFTHSDIQRMQ--ERERNK-GQ
Anolis carolinensis	Green anole	XM_008109785	XP_008107992	YTAFFTREDFRKMQ--ENEKNK-AQ
Python bivittatus	Burmese python	XM_015889024.2	XP_015744510	YLAFYSREDFRRMQ--EKEKNP-TQ
Pogona vitticeps	Central bearded dragon	XM_020794918	XP_020650577	YTALYSWEDFRRMQ--ERERNQ-AQ
Podarcis muralis	Common wall lizard	XM_028732029	XP_028587862	YLAFYTPDDFRKMQ--EKERNR-AQ
Pelodiscus sinensis	Chinese soft-shelled turtle	XM_014571642.2	XP_014427128.2	YLAFFTRSDIERMQ--ERERNK-AQ
Chelonia mydas	Green sea turtle	XM_027825953.2	XP_027681754	YLAFFTRSDIERMQLQEKERNK-AQ
**Amphibians**				
Cynops pyrrhogaster	Gaboon caecilian	XM_033918405	XP_033774296	YISFVSHNDATKMK--DRERNR-LQ
Ambystoma mexicanum	Axolotl			FLPIFTISESMRMQ--EKMRNN-AM
Cynops pyrrhogaster	Japanese fire belly Newt			FLPIFSPSDARRMQ--ERERNK-GM
Pleurodeles waltl	Iberian ribbed newt			FLPIFSPSDARRMQ--AKEKNR-AM
**Fish**				
Gadus morhua	Atlantic cod	XM_030375104	XP_030230964	HITFFSPREMMLM----KERDa#
Latimeria chalumnae	Coelacanth	XM_005995467	XP_005995529	FISFFSPSDMRRM--MEKEKSKALa
Cyprinus carpio	Common carp	LN590830		HIAFFSPKEMREL--REKEa
Oryzias latipes	Japanese medaka	XM_023955013	XP_023810781	HITFFSPKELLHM--RLQEQQEf##
Oncorhynchus mykiss	Rainbow trout	XM_036984493	XP_036840388	HFSFFSPKEMREM--KALQNKLa
Lateolabrax maculatus	Spotted sea bass	MH046054	AZM68775	HITFFSPKEMMLM----KEREa
Takifugu rubripes	Torafugu	XM_029826583	XP_029682443	HITFFSPKEMMVL----KQEQEa
Danio rerio	Zebrafish	NM_001386353	NP_001373282	HIAFFSPKEMREL----REKEa
Labrus Bergylta	Ballan wrasse			HITFFSPKEMMLM----KEREa*

Amino acids that differ from the human sequence are shown in red. The guinea pig genes shown in green is considered to be pseudogenized.

#, ## The small letter "a" and "f" indicate the C-terminal amidated and hydroxyl ternimi, respectively.

*Motilin structure is obtained from Zhou et al. (8)

GI motility patterns of the interdigestive and digestive periods are quite different for each mammal. A cyclic increase of GI motility called migrating motor complex (MMC) occurs in the interdigestive state with three phases: phase I (motor quiescent period), phase II (irregular and low-amplitude contraction period) and phase III (regular and high-amplitude contraction period) (6, 13–16). The function of MMCs is thought to include flushing out of the GI lumen mechanically and chemically for preventing bacterial overgrowth and receiving next meals (6, 17). After feeding, the cyclic motility pattern is suddenly disrupted and changed into irregular phasic digestive contractions, the amplitudes of which are close to those in the phase II. The duration of digestive contractions is more than 16 h and at the end, phase III-like contractions occur to completely remove the intraluminal contents, and GI motility changes into the interdigestive pattern (18). The well-known function of motilin is the GI motility activation of the stomach, small intestine and colon, and a typical example is the mediation of phase III of the gastric MMC in a fasting state in humans, dogs, and house musk shrews (*Suncus murinus* called *Suncus*) (6, 13–16, 19, 20). However, actions of motilin on motility of other digestive organs, such as the lower esophageal sphincter, gallbladder have been reported. In addition, other physiological effects of motilin on stimulation of gastric acid, pepsinogen, insulin and growth hormone release, and on food intake have also been reported (see another Section).

Motilin-induced actions are mediated by a G protein-coupled receptor (GPCR), GPR 38, called the motilin receptor (MLN-R), and which is mainly located on enteric neurons and smooth muscle cells of the GI tract in addition to its expression in the GI mucosa (21, 22). The presence of MLN-Rs in the central nervous system (CNS) has been also indicated (5, 23, 24).

**Figure 1:**
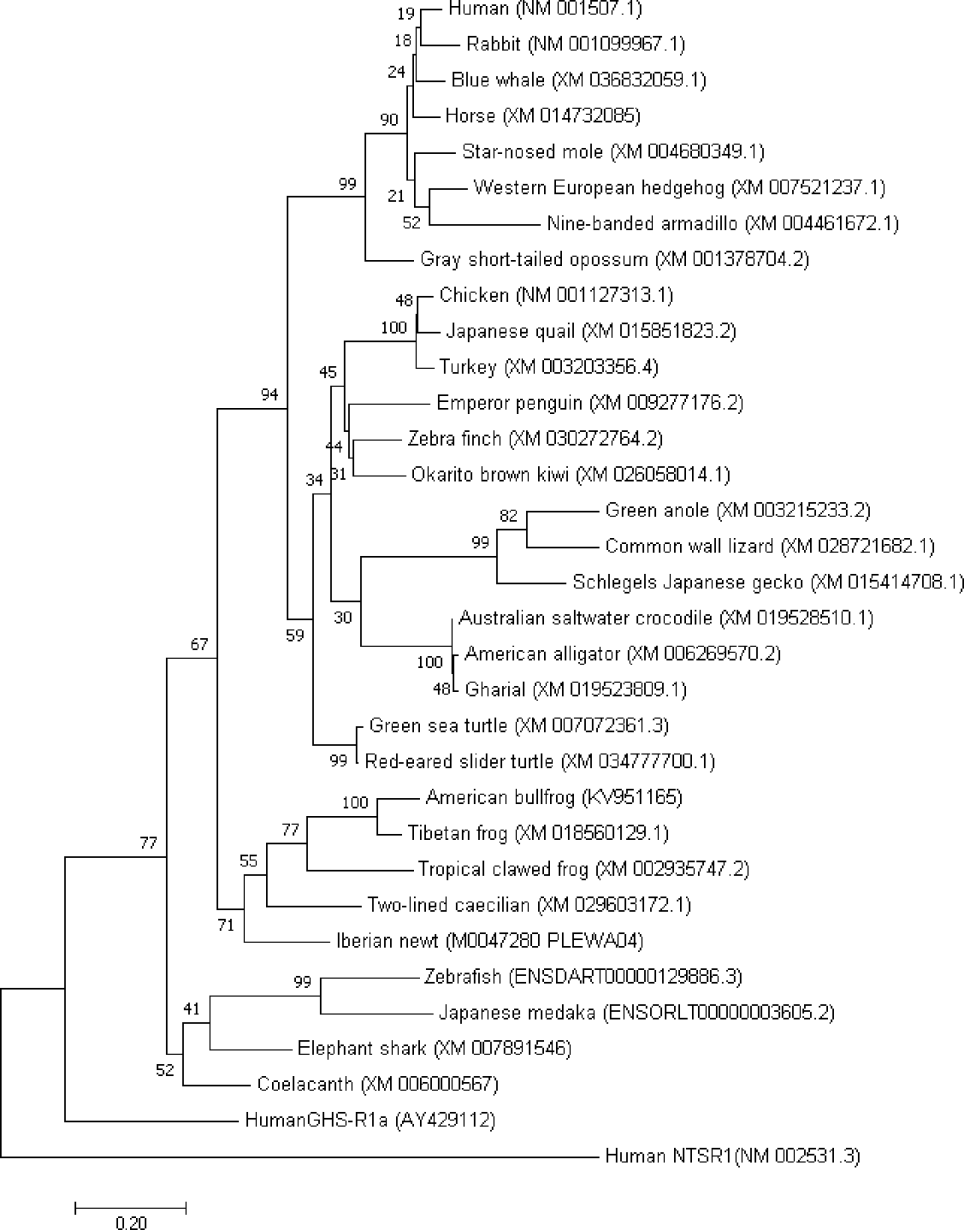
Molecular phylogenetic analysis of motilin receptor in vertebrates. The evolutionary history was inferred by using the Maximum Likelihood method based on the JTT matrix-based model. The tree with the highest log likelihood (-7475.17) is shown. The percentage of trees in which the associated taxa clustered together is shown next to the branches. Initial tree(s) for the heuristic search were obtained automatically by applying Neighbor-Join and BioNJ algorithms to a matrix of pairwise distances estimated using a JTT model, and then selecting the topology with superior log likelihood value. The tree is drawn to scale, with branch lengths measured in the number of substitutions per site. The analysis involved 33 amino acid sequences. All positions containing gaps and missing data were eliminated. There were a total of 264 positions in the final dataset. Evolutionary analyses were conducted in MEGA7.

The existence of motilin and its receptors in non-mammalian vertebrates such as birds, reptiles, amphibians, and fish has been demonstrated by identification of those mRNAs (**Figure 1**, **Table 1**), and comparative biological studies have been performed to clarify the functions of motilin in GI motility of these animals.

In this review, we focus on the results of biochemical, immunohistochemical and functional studies regarding motilin and MLN-Rs, and the roles of motilin in regulation of GI motility in mammals and non-mammalian vertebrates.

## Distribution of Motilin

### Peptide Distribution

Immunohistochemical approaches with human motilin-specific antiserum indicated that motilin-immunopositive (ip) cells are scattered in the mucosa of the upper intestine as open-type in humans (25), dogs (26), rabbits (*Leporinae Trouessart*, 27), sheep (*Ovis aries*, 28) and cattle (*Bos taurus*, 29). An open-type cell means that the endocrine cell is exposed to intestinal lumen and is activated by luminal chemicals including pH, whereas a close-type cell is a cell that is surrounded by other mucosal cells. In rabbits, motilin-ip cells are also found in the mucosa from the gastric antrum to distal colon, and the number of positive cells is the highest in the duodenum, moderate in the jejunum and low in other regions (27). In rodents, motilin-ip cells in the rat (*Rattus norvegicus*) intestine has been controversial: Smith et al. (30) failed to detect the motilin-ip cells for the human motilin antibody, whereas Vogel and Brown (31) and Sakai et al. (32) demonstrated the motilin-ip cells in the rat GI tract using anti-human and anti-chicken motilin antibodies, respectively. Recent genome-wide analyses have revealed that motilin and its receptor genes are pseudogenized in rodents including rats (10, 12). This discrepancy suggests that there are some systemic issues with immunohistochemical studies.

Immunohistochemical studies for non-mammalian vertebrates have shown by using anti-human motilin serum, and motilin-ip cells were detected in the duodenum but not in the proventriculus and gizzard of chickens (*Gallus gallus domesticus*, 33) and quails (*Coturnix japonica*, 34). Motilin-like immunoreactivity was detected in some reptiles (*Caiman latirostris, Caiman crocodilus, Egernia kingii*) but not in other reptiles (*Testudo graeca, Mauremys capsica, Lacetra lepida, Alligator mississippiensis*) or fish (*Tinca tinca*, *Ctenopharyngodon idellus*) (35–40). Because antibodies against human motilin were used in these studies, the sequence similarity with human motilin in these animals was suggested.

### Transcript Distribution

The highest expression of motilin precursor mRNA is seen in the duodenum of mammals, such as humans (41), monkeys (*Macaca mulatta*) (42), cats (*Felis catus*) (43), *Suncus* (44). Motilin precursor mRNA expression has not been investigated in the GI tract of birds, reptiles and amphibians. In fish, motilin precursor mRNA expression has been detected in the GI tract (8, 45). Brain such as the hypothalamus, hippocampus and cerebellum is an extra-intestinal expression of motilin precursor mRNA in some mammals (5, 42, 43).

## Characterizations of Motilin Sequence on Vertebrates

### Mammals

After the discovery of porcine motilin, motilin was isolated and its sequence was determined in humans (5, 46), rabbits (47), dogs (48), cats (49), monkeys (42), sheep (** 4) and *Suncus* (44). **Table 1** shows a comparison of the amino acid sequences in various vertebrates. Mammalian motilin is composed of 22-amino-acid residues. Structure-function relationship studies examined contraction and binding affinities of motilin fragments indicated the presence of three distinct regions in the motilin sequence, and these regions were suggested to have different functions. An *in vitro* study on rabbit duodenum contractile activity and displacement of [^125^I]-motilin binding in the rabbit antral membrane indicated that the N-terminal [1-7] is the minimum basic structure for binding and biological activity, the transit region [8-9] connects the N-terminal and C-terminal regions, and the C-terminal [10-22] forms an α-helix to stabilize the binding of the N-terminal and MLN-Rs (50, 51). On the other hand, an *in vivo* study in which GI motility was measured in conscious dogs indicated that the N-terminal is important for eliciting biological activities and that the middle and C-terminal portions are essential for preventing from the enzyme degradation (51). Although there are some differences in the sequence among mammals, the N-terminal region, which corresponds to the position at 1-7 (FV(M)PIFTY(H)) and the C-terminal region corresponding to the position at 14-18 (Q(R)EK(R)ER(Q) are highly conserved (**Table 1**).

It has been reported in rodents such as rats and mice that the motilin gene is pseudogenized (it used to code and generate motilin, but now it has lost the ability to produce motilin) (10). However, the motilin gene was deposited in the guinea-pig (*Cavia porcellus*), and its sequence has been estimated (FVPIFTYSEL RRTQEREQNKRL, 52). We attempted to re-examine the existence of the motilin gene (11). In our search of the Ensembl genome data, a guinea-pig motilin mRNA sequence encoding a 121-amino-acid precursor (ENSCPOT00000008024) was found, and a deduced mature sequence was estimated to be FIPIFTYSEL RRTQEREQNKGL (11, **Table 1**), in which two amino acids were different from that of Xu et al. (52). We tried to detect those transcripts using several primers sets, however, it could never be amplified (11), concluding pseudogenization of the motilin gene in guinea-pigs.

### Non-Mammals

Motilin has been identified in several avian species. Motilins of chicken, turkey (*Meleagris gallopavo*) and pheasant (*Phasianus colchicus versicolor*) and kiwi (*Apteryx rowl*) consist of 22 amino acids as in mammals, whereas the Bengalese finch (*Lonchura striata domestica*) and Golden eagle (*Aquila chrysaetos chrysaetos)* have 23 amino acids, and Rock pigeon (*Columba livia*) has 25 amino acids (**Table 1**). Motilins in Galliformes including chickens, pheasants, turkeys, and quails show a high homology with chicken motilin with a difference in only one amino acid. Three amino acids in finch and kiwi motilins are different from those in chicken motilin (**Table 1**). Therefore motilin sequences in avian species are highly conserved compared with those in mammals with diversified motilin sequences. When the motilin sequence of birds is compared with that of humans with focus on the N-terminal [1-10] and C-terminal [11-22], the homology of the C-terminal is high (from 83% to 92%) compared with the homology of the N-terminal (from 40 to 50%), suggesting a functional significance of conserved C-terminal sequence (**Table 1**).

**Figure 2:**
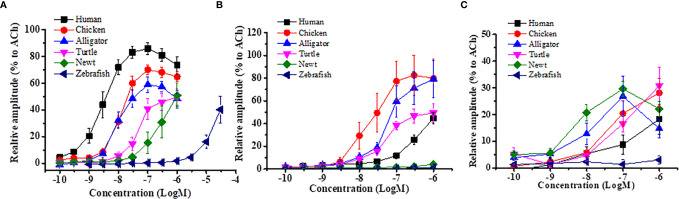
Comparison of contractile efficacy of different vertebrate motilins in isolated muscle strips from rabbit duodenum, chicken ileum and Japanese fire belly newt stomach. Isolated GI muscle strips from each animal were incubated in an organ bath containing bubbled physiological salt solution. Motilins were applied in the organ bath and evoked muscle contractions were measured by a force-transducer. Using this equipment, GI muscle-contracting actions of human, chicken, alligator, turtle, newt and zebrafish motilins were compared in the isolated rabbit duodenum **(A)**, chicken ileum **(B)** and Japanese fire belly newt stomach **(C)**. The symbols indicate concentration-response curves for the six motilins (human, chicken, alligator, turtle, newt and zebrafish). The Y axis indicates the relative amplitude of contraction normalized by the response of 10^-4^ M acetylcholine. Each symbol indicates the means ± SEM of results of at least five experiments. Homologous motilin showed the strongest response in respective GI strips (rabbit duodenum *vs.* human motilin; chicken ileum *vs.* chicken motilin; newt stomach *vs.* newt motilin).

Motilin in reptiles is also composed of 22 amino acids but it has a different amino acid at position 1 of the N-terminal (**Table 1**). Reptiles are divided into four orders: *Testudines* (turtles), *Sphenodontia* and *Squamata* (lizards and snakes) and *Crocodilia* (alligators). In mammalian and avian motilins, the amino acid at the N-terminal end begins with phenylalanine(F), but this depends on species in reptiles, i.e., alligator has F, but turtle, snake and lizard have tyrosine(Y). Homology between alligator/crocodile and human motilins is relatively high (73%), but those between turtle and human motilins, and between snake and human motilins are only 50% and 36%, respectively, suggesting that the motilin genes would have diverged dramatically in reptiles. Alligators are reptiles that are closely related to avian species (53) and that may be a reason for the high similarity of motilin sequence among mammals, birds and alligators. As seen in avian motilins, also in reptile motilin, the homology of the C-terminal region is high (from 58% to 92%) compared with the homology of the N-terminal region (snake, 0%; lizard, 10%; turtle, 20%; alligator, 50%). This high similarity of the C-terminal region of motilin, what does it mean? Because the N-terminal region is considered to be essential for motilin biological activities in dogs and rabbits (50, 54), low homology of the N-terminal region in reptiles might affect the biological activity in the mammalian GI tract. In fact, we found that turtle and alligator motilins cause contraction of the rabbit duodenum, but the affinity and amplitude of turtle motilin are considerably low compared with those of alligator, chicken, and human motilins (**Figure 2A**). This indicates the significance of the N-terminal sequence for GI-stimulating activity of motilin in mammals.

Amphibians consist of anura, urodela and dermophiidae orders. There has been no reports for identification of motilin, but we recently found urodelan newt motilin sequence by a BLAST search of a database in the Japanese fire belly newt (fire belly newt, *Cynops pyrrhogaster*) (http://antler.is.utsunomiya-u.ac.jp/imori/) and Iberia newt (*Pleurodeles waltl*, http://www.nibb.ac.jp/imori/main/) (**Table 1**). The motilin sequences of the fire belly newt and Iberia newt consist of 22 amino acids and are the same at the N-terminal [1-14]. The N-terminal sequence (FLPIF) is identical to that of alligators and is close to that of humans (FVPIF). We also searched axolotl database (*Ambystoma mexicanum*, http://ambystoma.uky.edu:4567), and found that the homology of fire belly newt and axolotl motilins to human motilin was 59% (13 of 22 amino acids being same). On the other hand, Gaboon caecilian (*Geotrypetes seraphini*), a species of amphibian (dermophiidae) has a different sequence from those of axolotl and newt, and the amino acid at the N-terminal end begins with tyrosine(Y) as seen in turtle, snake, and lizard (**Table 1**). Homology of amphibian motilin to human motilin is higher in the C-terminal [11-22] sequence (42-57%) than that of N-terminal [1-10] sequence (0-50%) as with avian and reptile motilins. We tried to examine the contractile activity of newt motilin in isolated rabbit duodenum and chicken ileum and found that newt motilin induced a small contraction in the rabbit duodenum but no response in the chicken ileum (**Figure 2**), while newt motilin showed a high responsiveness in the newt stomach (**Figure 2**). These results suggest that binding affinity of amphibian motilin to mammalian and avian MLN-Rs is very low due to the critical sequence differences, and amphibian motilin has an ability to bind MLN-R and to cause GI contraction of amphibians itself.

Motilin peptides have been identified in various fish, and their amino acid sequences are quite different from those of other vertebrates (**Table 1**). The N-terminal end of motilin in most fish begins with histidine (H), and the amino acid sequence varies from 17 to 21 residues depending on the species. The N-terminal [1-10] of fish motilin is well conserved. When the sequence was compared with human motilin, the homology of the N-terminal [1-10] region is 20% and that of the total sequence is only 24% (4 of 17 amino acids). Intriguingly, motilin sequence of the coelacanth (*Latimeria chalumnae*), relative of tetrapod, is different from other fish motilins: the coelacanth motilin consists of 22 amino acids as in most vertebrates and starts with phenylalanine (F) as birds and mammals (**Table 1**). It may be that the molecules retain vestiges of the process of evolving into land animals.

### Summary of Structural Characterizations of Motilin

A highly conserved N-terminal sequence starts with phenylalanine (F) is thought to be essential for biological activity in mammalian/avian motilins. Reptile motilin is just in the transition stage to mammalian/avian type. In alligators, lineage of reptile motilin may have evolved under different evolutionary pressures to modify sequence of motilin close to the mammalian/avian type. On the other hand, sequences of fish and amphibian motilins quite differ from those of mammalian/avian motilins. In the molecular evolution of motilin, there may have been a major event at the time the reptiles emerged.

Comparison of sequence of vertebrate motilin indicates that C-terminal sequence is more markedly conserved than that of the N-terminal sequence. It suggests that the C-terminal might have a function other than stimulation of GI motility mediated by the N-terminal. C-terminal portion is thought to form α-helix and to stabilize the binding of motilin molecule with MLN-R and to prevent its degradation by enzymes (50, 51), and it has been reported to contribute enhancement of desensitization, phosphorylation, and internalization of MLN-R (55), probably due to formation of stable binding to MLN-R. Possibility of other unknown functions of the C-terminal conservation of motilin cannot be ruled out.

## Motilin Receptor

### Agonists and Antagonists

At the beginning of motilin study, radioligand binding studies showed the presence of high affinity binding sites saturated by motilin in membrane preparations from human, rabbit, cat and canine GI tracts and this binding site was proposed as the MLN-R (56–60). In the GI tract, MLN-Rs were thought to be present on both muscle cells and enteric neurons (56, 57, 60).

Erythromycin, a commonly used macrolide antibiotic, has been known to have GI side effects (vomiting and diarrhea) (61). Itoh et al. (62) and Inatomi et al. (63) reported that erythromycin and its derivative caused GI contraction of the conscious dogs similar with motilin. *In vitro* studies also indicated that erythromycin contracted the rabbit duodenum as did motilin (64, 65). Binding studies clearly indicated that erythromycin bound to MLN-R and displaced a labelled motilin binding (64, 66, 67). Therefore, it was thought that macrolide antibiotics including erythromycin could bind to MLN-Rs and acted as motilin agonists causing GI contractions. These compounds are termed motilide from the two words “motilin” and “macrolide”.

In early physiological studies, anti-motilin serum or motilin-induced MLN-R desensitization was used to confirm involvement of motilin, but those approaches also caused non-specific actions. Therefore, the need for specific antagonists for the MLN-R has increased to perform detailed physiological studies. In 1995, two MLN-R antagonists, [Phe^3^, Leu^13^] porcine motilin and GM109, were reported (68, 69). Later, MA2029, a 10-times potent and selective MLN-R antagonist was also reported (70). Using these MLN-R antagonists, involvement of endogenous motilin in the phase III of gastric MMC initiated in fasted dogs or *Suncus* was confirmed (71, 72).

### Structural Characteristics of MLN-R In Vertebrates

The molecular structure of MLN-R was first identified in the human stomach as an orphan GPCR (GPR38) (66, 73). GPR38 is highly expressed in the human duodenum and colon. A study using mutants of MLN-R indicated that motilin and erythromycin share a common binding site in the third transmembrane (TM3) region (74). A photoaffinity labeling study also indicated that the first and second extracellular loop domains located close to TM3 are important for binding of motilin (75).

Because many studies have been performed using dog and rabbit GI tracts, MLN-R cloning has firstly been conducted in these animals. The homologies of the deduced dog and rabbit MLN-Rs to the human MLN-R are 84% and 71%, respectively (76, 77). Later, Suzuki et al. (78) reported the *Suncus* MLN-R, and showed high homology (76%) to the human MLN-R, and the affinity of the *Suncus* MLN-R for MLN-R agonists was comparable to that of the human MLN-R.

The amino acid sequence of the human MLN-R showed a relatively high homology with that of growth hormone secretagogue receptor 1a (ghrelin receptor) of humans (52%) and *Suncus* (42%) (66, 78). When the amino acid sequences of seven transmembrane domains were compared, the homology between human MLN-R and ghrelin receptor further increases (86%). Therefore, MLN-R is considered to be a sister receptor with ghrelin receptor (79).

However, ghrelin cannot activate MLN-R of the rabbit stomach (80), canine or human MLN-Rs expressed on CHO cells (77). In an *in vivo* study with dogs, it was found that the ghrelin decreased the phase III of gastric MMC different from the action of motilin (14). Inconsistent of actions induced by ghrelin and motilin suggests that motilin cannot stimulate the ghrelin receptor, although there is some amino acid sequence similarity in the two receptors. Similarly ghrelin also cannot act on MLN-Rs.

In research for non-mammalian MLN-Rs, Yamamoto et al. (81) firstly characterized the chicken MLN-R identified in the duodenum. The chicken MLN-R consists of 349 amino acids and showed 59% sequence identity to the human MLN-R. The chicken MLN-R expressed on HEK293 cells responded to human and chicken motilins, but chicken motilin has higher affinity than human motilin as was shown in an *in vitro* contraction study (82). The low homology of the chicken MLN-R to the human MLN-R might explain the low contractile affinity of human motilin (**Figure 2**), and the ineffectiveness of erythromycin or MLN-R antagonists (GM109 and MA2029) (82, 83). In amphibians, human motilin causes a contraction of the upper intestine of the bullfrog and tropical clawed frog (*Xenopus tropicalis*) and of the stomach of the black spotted pond frog (*Pelophylax nigromaculatus*) (84, 85), suggesting the presence of MLN-Rs in GI tract at least in these frogs. Erythromycin and GM109 were ineffective in the bullfrog intestine, suggesting a different structure of amphibian MLN-R from the human MLN-R (85). In the Ensembl database search, we found a candidate MLN-R for the tropical clawed frog (ENSXET00000013318), but its ligand, endogenous motilin, could not be found (**Table 1**). This indicates that anuran amphibians have lost only motilin for some reason during their evolution without losing the MLN-R. The retained MLN-R is thought to function for exogenous motilin. Another endogenous agonist may be acting on this MLN-R.

The zebrafish and spotted sea bass MLN-Rs have been reported (8, 45). Zebrafish MLN-R consists of 345 amino acids and shares 47% identity to the human MLN-R. The zebrafish MLN-R expressed on HEK293 cells was activated by homologous zebrafish motilin with an increased intracellular Ca^2+^ concentration, whereas human motilin did not activate at least at a concentration of 100 nM (86). This indicates a strong species-specific relationship of the ligand-receptor interaction in the fish motilin system, and this can be expected from the sequence of motilin, which is unique to fish.

### Phylogenetic Tree of MLN-R in Vertebrates

The phylogenic tree created by amino acid sequence of MLN-Rs indicates two main branches have evolved: one group (group A) is composed of tetrapods including mammalian, avian, reptile and amphibian MLN-Rs and the other group (group B) contains fish MLN-Rs (**Figure 1**). Group A can be divided into two clades: terrestrial (mammals, birds and reptiles) type and semi-aquatic (amphibian) type. The clade of the avian/reptile MLN-Rs can be further divided into three, and alligator/crocodile MLN-Rs is included in the same umbrella with the avian clade, as in the case of motilin structure. Group B may have characteristics that match the aquatic inhabiting nature of fish.

## Regulation of Motilin Release

The effects of bioactive substances and nutrients on the release of motilin are summarized in **Table 2**. Cyclic increases of plasma motilin with 100-min intervals have been reported in fasting periods in humans, dogs, and opossums (6, 16, 104). This cyclic increase is inhibited by feeding, and motilin stays low level during the digestive state. Infusions of nutritional factors such as glucose and amino acids in the duodenum decrease motilin release (90), indicating that feeding-related decrease in motilin release might be caused by sensing digestive nutrients in the duodenum. However, the effects of fat are controversial: no effect (90, 96) and stimulatory (95) (**Table 2**). In humans, feeding caused a transient increase in plasma motilin concentration, and both cerebral excitation by feeding and gastric distension by meals were thought to participate in this motilin increase (103).

**Table 2:** Regulatory stimulants for endogenous motilin release in mammals.

	Responses		
	Increase	Decrease	No effect
Bioactive substances	Acetylcholine [direct action]{dog} (87, 88)	Ghrelin {dog} (14)	CCK [in vitro, in vivo] {dog} (87, 89)
	Bombesin [direct action]{dog} (88)	Somatostatin {dog} (88, 90)	Gastrin [in vivo] {dog} (89)
	Serotonin [indirect ACh release]{dog} (91, 92)	Insulin {dog} (93)	Secretin [in vitro, in vivo]{dog} (87, 89)
	Motilin [indirect serotonin and ACh release]{dog} (91)	α-adrenerigic receptor {dog} (88)	Serotonin [in vitro] {dog} (87)
	Prostaglandin E2 [indirect ACh release]{dog} (94)	Pancreatic polypeptide {human} (20)	
Nutrients	Fat {dog} (95)	Feeding {dog} (19)	Fat {human, dog} (90, 96)
		Glucose {dog} (90)	
		Amino acid{dog} (90)	
Chemicals	Alkalinization {dog, suncus} (97–99)	Acidification {dog} (100)	Alkalinization {human} (96)
	Acidfication {human, dog, pig, suncus} (96, 98, 99, 101, 102)		
Mechanics	Increase in luminal pressure {dog} (17)		Vagotomy {dog} (93, 95)
	Gastric distension {human} (103)		

Pharmacologically, the cyclic increases of motilin are inhibited by atropine or hexamethonium, and a vagus nerve stimulation causes an atropine- or hexamethonium-sensitive increase in motilin release (105–108). Injection of a muscarinic agonist, carbachol into the duodenal artery of anesthetized dogs increased motilin release, and the increase was inhibited by atropine but not by tetrodotoxin or hexamethonium (106). Therefore, a neural network involving ganglionic nicotinic receptors and muscarinic receptors on non-neural tissues could mediate the motilin release. The muscarinic receptor-mediated motilin release has been demonstrated in intestinal mucosal motilin-producing cells of dogs (87).

Stimulation of vagus nerves increased plasma motilin concentration, but chronic vagotomy and blockade of vagus nerves by cooling had no effects on motilin release in dogs (93, 95, 109), suggesting that motilin release is regulated by both vagal and non-vagal cholinergic pathways.

Motilin and erythromycin induce motilin release through activation of positive feedback mechanism mediated by the 5-hydroxytryptamine3 (5-HT_3_) receptor and nicotinic and muscarinic receptors. Motilin stimulates the release of 5-hydroxytryptamine (5-HT) and acetylcholine (ACh), and 5-HT induces ACh release from enteric cholinergic neurons. Finally, ACh activates muscarinic receptor on the motilin-producing cells in the duodenum (87, 91, 110).

In dogs, ghrelin decreases motilin release, and cyclic changes in plasma ghrelin are reversal to cyclic changes in plasma motilin (A peak of ghrelin is corresponding to bottom of motilin and the bottom of ghrelin is a peak of motilin). At least in dogs, ghrelin regulates the release of motilin although the mechanisms of cyclic changes in ghrelin were not clarified (14). In humans, however, plasma ghrelin does not fluctuate and does not affect motilin release (111, 112), suggesting a dog-specific regulation of motilin release by ghrelin.

Bombesin, prostaglandin E_2_ (PGE_2_) and 5-HT stimulate the release of motilin, but somatostatin, insulin, and noradrenaline (α-adrenoceptor) decrease (**Table 2**). Investigation in dispersed motilin-producing cells in dogs indicated that there are excitatory muscarinic and bombesin receptors and inhibitory somatostatin and α-adrenoceptor receptors on the motilin-producing cells (87, 88). Therefore, PGE_2_ and 5-HT are thought to stimulate ACh release from the cholinergic neurons and to act on the motilin-producing cells indirectly (87, 88, 94).

Duodenal pH influences gastric motility and motilin release. Dryburgh and Brown (97) reported that duodenal alkalization increased gastric motor activity in association with increased motilin concentrations in dogs. Three phasic changes in duodenal pH (a weak acid period, strong acid period and alkaline period) observed in dogs were associated with three types of gastric contractions (the digestive, intermediate, and interdigestive MMC) (113). An association between duodenal pH and gastric motility has also been reported in humans. Woodtli and Owyang (114) found that duodenal pH changed from 2 to 7.5 during the onset of phase I to phase III, and that pH was maintained at alkaline from late phase II to phase III of the gastric MMC. Acidification-induced motilin release was also observed in the isolated perfused pig duodenum (101). These studies have indicated that both duodenal acidification and alkalinization stimulate motilin release and induce GI contraction like phase III (**Table 2**). The mechanisms by which opposite pH stimulations cause almost the same gastric contractions through motilin release have been investigated in *Suncus*. Mondal et al. (99) examined the association of duodenal pH and gastric phase III contractions by motilin and reported the mechanisms for motilin release by a change in duodenal luminal pH as follows: acidification of the duodenal lumen by gastric acid stimulates the synthesis of PGE_2,_ which decreases the release of gastric acid and simultaneously increases 5-HT release from enteric 5-HT neurons and mucosal enterochromaffin cells; 5-HT activates the release of bicarbonate from mucosal cells by activation of the 5-HT_4_ receptor and the released bicarbonate increases the luminal pH; finally, alkalinization of the lumen stimulates the release of motilin to cause the gastric contraction, although the mechanisms of motilin release by luminal alkalinization have not been clarified. The interval of appearance of gastric phase III of the MMC is a required time that duodenal acidification finally causes alkalinization in the duodenum through the pathway including PGE_2_, 5-HT/5-HT_4_ receptor and bicarbonate. The increase in the 5-HT concentration in the duodenal lumen by PGE_2_ is also thought to contribute to the initiation of duodenal MMC (99).

Takahashi (17) reported another idea of periodic release of motilin using dogs as model animals. At first, in phase I, gastric, pancreatic and biliary juices increase luminal pressure of the duodenum and the increase in pressure stimulates the release of 5-HT from the enterochromaffin cells by mechanoreceptor. There is a positive circuit between 5-HT release and increase in luminal pressure. 5-HT stimulates the duodenal pressure and the pressure increases the release of 5-HT. Duodenal 5-HT increases duodenal pressure corresponding to intestinal phase II and III contractions, and the increased duodenal pressure stimulates the release of motilin. The released motilin further increases the release of 5-HT, and the increased 5-HT finally stimulates vagal afferent neurons to cause gastric phase III through the 5-HT_3_ receptor on the afferent terminals (115). Activation of enteric cholinergic neurons by neural MLN-R also contributes to initiation of the gastric phase III contraction. Therefore, after appearance of the gastric phase III contraction, 5-HT in enterochromaffin cells is exhausted and it takes times to refill with 5-HT. This “time” is considered to be the interval of periodic release of motilin and the motilin-induced gastric phase III of the MMC. Augmentation of duodenal motility causing an increase in luminal pressure might be a stimulant for motilin release (17).

The regulation of motilin release and the corresponding GI motility have been performed in the dogs, humans and Suncus (**Table 2**). Species-related differences including non-mammalian vertebrates on the regulation of motilin release should be examined in future.

## GI Motility-Stimulating Actions in Mammals

**Figure 3:**
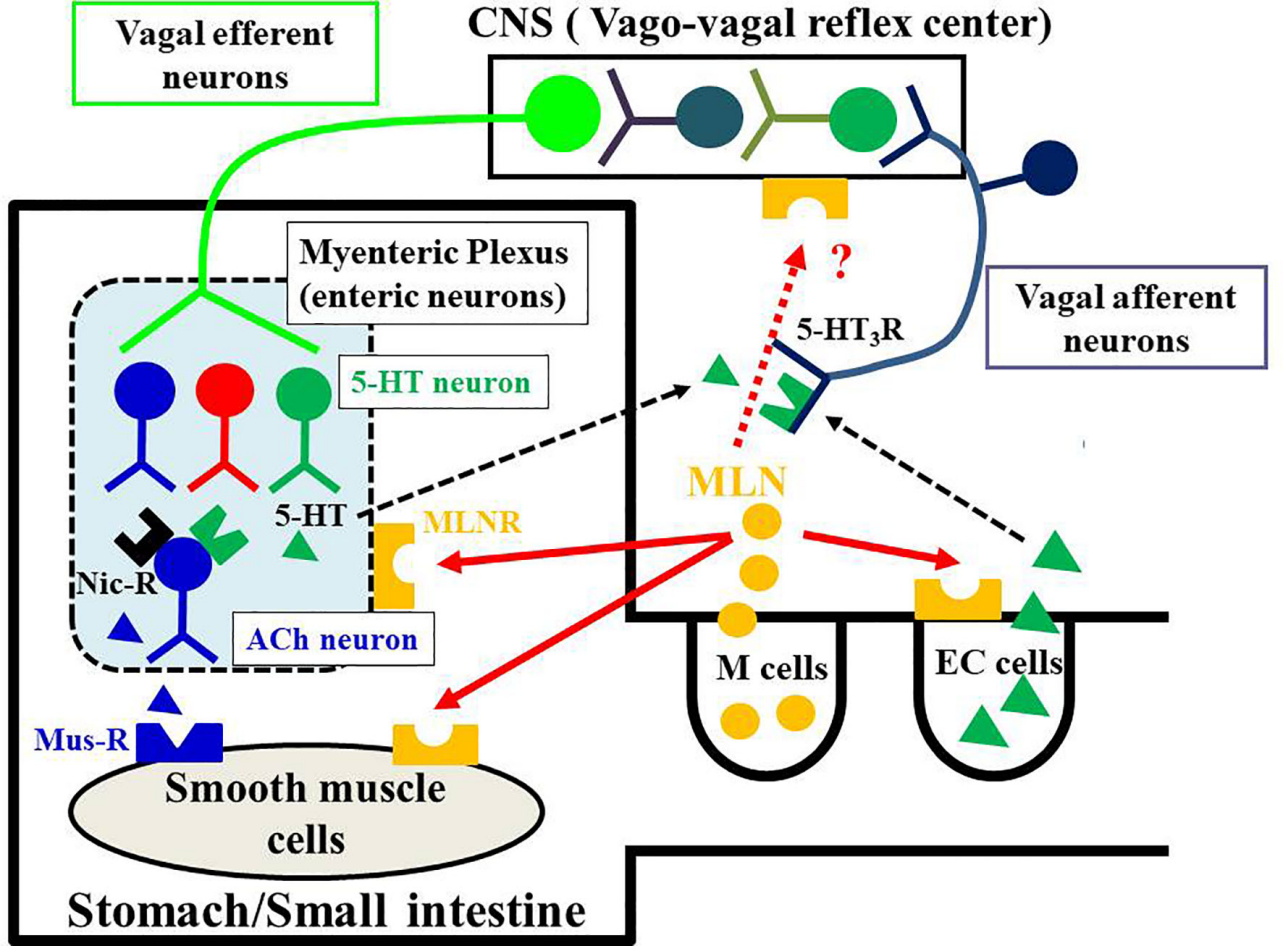
Potential mechanisms of motilin-induced GI motor-stimulating actions. Motilin is synthesized in the M cells of the upper GI tract and is released by various stimuli, including mechanical, chemical, and biological. The released motilin causes GI motility-stimulating actions through motilin receptors (MLN-Rs) located on enteric neurons and smooth muscle cells. Neural pathways in the enteric nervous system are complex. Motilin stimulates neural pathways including cholinergic nicotinic receptors (black), adrenergic receptors, serotonin (5-HT) receptors and NO neurons, and finally acetylcholine (ACh, blue triangle) released from cholinergic neurons (blue) acts on muscarinic receptors (Mus-R) on smooth muscle cells to cause contraction of stomach and upper intestine. Results of experiments in conscious animals (dogs, humans and *Suncus*) indicate that motilin stimulates the release of 5-HT from enteric serotonergic neurons (green) and 5-HT (green triangle) activates both enteric cholinergic neurons and the vago-vagal reflex pathway through activation of the 5-HT_3_ receptors on enteric neurons and afferent vagal terminals. The stimulation of vagus efferent neurons activates neurons in the myenteric plexus to cause contraction of stomach. Since MLN-R is also present in the intestinal mucosa, it is possible that motilin acts on enterochromaffin cells (EC cells) to release 5-HT. The 5-HT originating from EC cells could also act on enteric neurons and the vagus afferent terminals. The contribution of these mechanisms might be different depending on the species, regions, and experimental conditions. The vago-vagal reflex pathway has been demonstrated mainly in the stomach but not in the small intestine. The MLN-R is also expressed in the CNS, but its functional roles in stimulating GI motility is unknown.

The effect of motilin is different depending on animal species, GI regions and experimental conditions (*in vivo* and *in vitro*). In *in vivo* experiments, changes in intraluminal pressure, muscle contractility or muscle myoelectric activity were measured using conscious or anesthetized animals. Measurements of gastric emptying and intestinal transit are other ways to evaluate GI motility. Under these experimental conditions, extrinsic and intrinsic neural networks of the GI tract are intact, and the afferent-to-efferent autonomic nervous reflex pathways are also intact. On the other hand, isolated GI smooth muscle preparations used in *in vitro* study are cut off from extrinsic innervation from brain and sensory innervation connecting to brain. However, enteric neurons in the myenteric and submucosal plexuses are intact and functional. These enteric neurons are able to stimulate electrically. In *in vitro* experiments, on the other hand, the local actions of motilin on smooth muscle cells and enteric neurons can be examined. Based on the results of functional studies mainly used dogs, rabbits and *Suncus*, the mechanisms of GI motility-stimulating actions by motilin are divided into three pathways (6, 7, 71, 99, 116, 117) (**Figure 3**): (i) the action on MLN-Rs located on smooth muscle cells; (ii) the action on MLN-Rs located on enteric neurons although detailed neural networks have not been proven, as a result, ACh released from cholinergic neurons causes contraction through the muscarinic receptor; and (iii) the activation of the vago-vagal reflex pathways followed by stimulating vagal efferent neurons connecting to the enteric neurons. The presence of 5-HT_3_ receptors has been demonstrated in the terminals of vagus afferent neurons (115), and motilin-induced contraction in the vagus-intact stomach, but not in the vagotomized stomach, was decreased by a 5-HT_3_ receptor antagonist (94). Thus, motilin is thought to stimulate the release of 5-HT from enteric neurons and enterochromaffin cells, and the released 5-HT activates the 5-HT_3_ receptors on the terminals of vagal afferent neurons. Contribution of three mechanisms to the motilin-induced GI contraction is different from animal species and GI regions. Although expression of MLN-Rs in the CNS has been reported (5, 23, 118), contribution of motilin and MLN-Rs in the CNS to the GI motility-stimulating actions might be excluded because intrathecal or intracerebroventricular injection of motilin failed to cause GI contraction in dogs (119), and motilin is a hydrophilic peptide and not able to penetrate the blood-brain-barrier.

Motilin and erythromycin cause successive phasic phase III-like contractions of the GI tract and accelerate gastric emptying and intestinal transit (6, 13, 62, 120). The mechanisms for eliciting rhythmic contractions consisting of contraction and relaxation are estimated as follows. At the smooth muscle cell level, MLN-R is coupled with G_q/11_ linked to phospholipase C that synthetizes IP_3_ and diacylglycerol. IP_3_ stimulates the release of Ca^2+^ from intracellular store and the influx of extracellular Ca^2+^. Then increase in intracellular Ca^2+^ evokes both muscle contraction (121, 122), and muscle relaxation through activation of Ca^2+^-activated K^+^-channels (123). On the enteric neuron levels, it is known that motilin acts on both excitatory cholinergic and inhibitory nitrergic neurons in the rabbit (124), *Suncus* (71) and chicken GI tracts (125). At the vago-vagal reflex level, vagal efferent neurons innervate both excitatory and inhibitory neurons in the myenteric plexus.

In the following sections, the effects of motilin on GI motility in each animal are described in detail.

### Dogs

Dogs have been used since the early days of motilin research because the size is suitable for surgical operations and for drawing blood samples several times.

Itoh et al. (13) reported that the GI motility patterns of dogs in the digestive and interdigestive periods are quite different. In the interdigestive period, i.e., a cyclic increase of GI motility consisting of phase I, phase II and phase III occurs in the stomach with an interval of 80-100 min and it propagates to the caudal direction. Therefore, the cyclic GI motility is called interdigestive MMC. Motilin caused a contraction similar to that of MMC in the canine stomach, and this contraction migrated in the direction toward the small intestine. On the other hand, there is no MMC in the digestive state, and motilin does not cause any motility changes in this state (13). In addition, Itoh et al. (19) demonstrated that the peak of plasma motilin concentration was associated with the occurrence of phase III activity. Phase III contraction has been demonstrated to be disrupted by anti-motilin serum, or a motilin receptor antagonist (72, 126). Therefore, motilin has been thought to be an endogenous regulator of phase III activity of the MMC in the fasting state. Although Lee et al. (126) showed interruption of the gastric MMC by treatment with anti-motilin serum, the MMCs in the distal intestine were resistant, suggesting that the mechanisms of gastric and intestinal MMCs are different and that motilin is not a meditator of intestinal MMC.

The mechanisms of motilin-induced contractions in dogs have been analyzed by autonomic drugs and denervation of vagus nerves. The motilin-induced gastric contractions were sensitive to atropine and hexamethonium, indicating the involvement of a neural pathway including nicotinic and muscarinic receptors. The involvement of vagus nerves in motilin-induced contractions has been also reported. A low dose of motilin stimulated GI motility through activation of the 5-HT_3_ receptors on the vagus nerves and vagal reflex pathway, but a high dose caused an atropine-sensitive GI contraction through activation of enteric cholinergic nerves independent of the vagus innervation (127, 128). Therefore, a physiological concentration of motilin stimulates enteric neurons both by direct and indirect actions through vagal afferents to vagal efferents pathway. Tanaka et al. (129) reported that the vagal nerves were not necessary for the initiation or coordination of fasting gastric MMC patterns but were involved in the modulation of the contraction pattern during gastric MMC. Taken together, the results indicate that motilin causes the phase III of gastric MMC and simultaneously modulates the frequency and amplitude of the MMC pattern through actions on the vago-vagal reflex pathway.

*In vitro* studies using the isolated canine antrum and duodenum indicated that canine motilin caused contraction at a very high concentration (130) and that porcine motilin was ineffective. An approximately 10,000-times higher concentration of canine motilin was necessary for contraction of the canine duodenum compared with the concentration for contraction of the rabbit duodenum (131, 132). A receptor binding study failed to detect specific motilin binding sites (60). Therefore, the isolated canine duodenum is insensitive to motilin due to the lack of MLN-Rs.

However, another *in vitro* study using the isolated vascularly perfused canine small intestine showed that intra-arterially injected motilin increased luminal pressure and that it was antagonized by tetrodotoxin, atropine and hexamethonium, indicating that motilin acts on the enteric preganglionic and postganglionic cholinergic nerves (133). Kellum et al. (134) showed cholinergically mediated release of 5-HT from enteric neurons and that the 5-HT_3_ receptor mediated the contractile actions of motilin in the canine jejunum. Similar involvement of 5-HT in the motilin-induced contraction was also demonstrated in the isolated perfused canine stomach. It has been shown that the neural pathway including α-adrenoceptors is involved in motilin-induced gastric actions (135). In addition, motilin had no effect on spontaneous contraction but increased the amplitude of electrically induced cholinergic contraction in isolated canine small intestine (136). These observations indicate that motilin can cause GI contractions *via* activation of enteric neurons in *in vitro*. Immunohistochemical and molecular biological studies indicated the presence of the MLN-Rs in the enteric plexus (21).

Taken together, the results in dogs suggest that motilin stimulates (i) vagal afferent neurons connecting to the vagal efferent neurons that synapse to enteric neurons through 5-HT/5-HT_3_ receptor and (ii) enteric neurons of myenteric plexus including adrenergic (α-adrenergic receptors), serotonergic (5-HT_3_ receptors) and cholinergic interneurons (nicotinic receptor), and that motilin finally releases ACh from cholinergic neurons, which causes contraction of the stomach, that is phase III of the MMC, although the arrangement of neural networks in the myenteric plexus has not been determined (17) (**Figure 3**).

Sanger et al. (12) suggested that the motilin system is related to the ability of vomiting. Application of motilin or erythromycin frequently caused vomiting in dogs (63, 128). Motilin might be mimic the vomit-related GI motility (retroperistalsis) in addition to the regulation of phase III of the MMC in interdigestive periods. Similar to motilin-induced contraction, 5-HT, the 5-HT_3_ receptor and afferent terminals of the vagus nerves have been shown to be involved in the vomiting caused by the anti-cancer drug cisplatin (137). Therefore, the neural pathway involved in motilin-induced gastric contraction is partially involved in anti-cancer drug induced vomiting mechanisms.

### Rabbits

Strunz et al. (9, 138) found that the rabbit GI tract was sensitive to motilin. Considerable GI region-dependent different responsiveness was found: the upper GI tract including gastric antrum, duodenum and jejunum was sensitive to motilin, but the ileum was insensitive (139, 140). In the duodenum, the contraction induced by motilin was not decreased by atropine and tetrodotoxin (9, 139, 140) and the responses evoked by neural stimulation were not modified by motilin (139). These results suggest a direct action of motilin on smooth muscle. In a study using a dispersed rabbit antral smooth muscle cells, motilin caused the shortening of the isolated cells (141). Motilin binding sites were demonstrated in dispersed muscle cells (142) and in smooth muscle membrane fractions (143). These results indicate that MLN-Rs are located on the smooth muscle cell membrane as myogenic receptors. However, other studies showed enhancement of neural contractions and stimulation of the release of [^3^H]-ACh by motilin, indicating that motilin also acts on enteric neural MLN-Rs (117, 144). GI region-dependent distributions of myogenic and neural MLN-Rs have been demonstrated. Poitras et al. (145), Van Assche et al. (143) and Miller et al. (59) reported the results of [^125^I]-motilin binding studies using neural synaptosomes and smooth muscle membranes obtained from the antrum, duodenum, and colon. Although both smooth muscle and neural MLN-Rs exist in each region, MLN-Rs are predominantly distributed in the neural fraction in the gastric antrum while those are abundant in the smooth muscle fraction in the duodenum and colon. Although the binding affinities for labelled motilin on smooth muscle and neural binding sites are comparable, the affinities of some synthetic MLN-R antagonists for neural motilin binding sites are higher than those for smooth muscle motilin binding sites (59). Poitras et al. (145) reported that the affinities of motilin and erythromycin were significantly different in the antral neural receptor fraction and the duodenal smooth muscle receptor fraction. However, the details of these differences, i.e., subtypes of MLN-R, have not been clarified, and only one MLN-R has so far been cloned in rabbits (76).

There have not been many *in vivo* studies on GI motility in rabbits since rabbits eat small meals frequently and their stomachs will never be empty, i.e., “fasted until death”, suggesting that rabbits do not have fasting period and interdigestive GI motility. An *in vivo* study in which myoelectric activity of the GI tract was recorded in conscious rabbits indicated that the migrating myoelectric activity consisting of three phases originated from the proximal jejunum, not the stomach and duodenum, being different from that in dogs, and that the myoelectric activity appears in both feeding and fasted rabbits at almost the same intervals (146). The plasma motilin concentration has not been measured in rabbits, but Guerrero-Lindner et al. (147) examined the effect of motilin on the GI electric activity. They found that motilin did not affect the antral electric activity but increased duodenal and jejunum activities. However, the motilin-induced activity did not propagate downward and was not followed by a quiescent period like phase I, being different from the pattern of spontaneous myoelectric activity, suggesting that motilin is not likely to be a physiological regulator of the migrating myoelectric activity in rabbits. Atropine, hexamethonium and ondansetron did not change the motilin-induced myoelectric activity in rabbits in contrast to the results in dogs (147), indicating that motilin acts directly on the smooth muscle MLN-Rs (**Figure 3**). However, in *ex vivo* intestinal preparations (stomach and upper intestine were isolated together and incubated in an organ bath), motilin caused migrating motor activity in the duodenum and these activities were decreased by atropine, indicating that the motilin-induced actions are of cholinergic neural origin (148). One of the discrepancies between *in vivo* and *ex vivo* studies can be explained by the concentration of motilin applied intravenously. In conscious dogs and *Suncus*, motilin (0.1 µg/kg, i.v.) was used to initiate phase III-like activity, which was a neural origin (13, 15), while high concentrations of motilin (0.6 µg/kg-1.5 µg/kg, 147) used in the rabbits were possible to act on smooth muscle MLN-Rs and myogenic actions masked the neural actions. Concerning rabbit myoelectric activity, Marzio et al. (148) reported the occurrence of a spontaneous myoelectric complexes originating from the duodenum in an *ex vivo* rabbit intestinal preparation, in agreement with the results of *in vivo* studies (146, 147). In the *ex vivo* study, motilin induced MMCs in both the gastric antrum and duodenum, but spontaneous myoelectric activities were only elicited in the duodenum regardless of the absence or presence of motilin in the organ bath (148). Therefore, although the possibility of contribution of endogenous motilin to the spontaneous migrating myoelectric activity in the *ex vivo* study cannot be completely excluded, it is suggested that motilin does not initiate the physiological migrating myoelectric activity in the rabbit duodenum but possibly regulates the appearance of this activity.

Motilin-induced GI motor-stimulating actions in rabbits have been also examined under an anesthetized condition. It was found that motilin caused contractions of the stomach and colon but not the ileum (140). The high responsiveness of the isolated colon to motilin (140) and high density of motilin binding sites (149) found in *in vitro* studies may reflect the results of the *in vivo* study. Mitemcinal, an MLN-R agonist was reported to increase the defecation in the conscious rabbits (150). Rabbits belong to the order Lagomorpha, not Rodentia, and are coprophagous grass-eating animals with a property of hindgut fermentation. The regulation of colonic motility is important for rabbits and motilin might be regulator of the colonic motility.

Although rabbits have been widely used in studies for GI motility-stimulating actions of motilin, the physiological roles are still not well understood. To determine the roles of motilin in rabbit GI motility, a study in which measurement of plasma motilin concentration and an *in vivo* contraction study using a physiological dose of motilin are necessary. Since rabbits do not have an interdigestive GI motility state like that in dogs due to their eating behavior, motilin might have different roles in regulation of GI functions including motility, absorption and secretion. Motilin has been shown to regulate amino acid absorption in the rabbit intestine (151).

### Rodents

It has been known for a long time that motilin does not cause contraction in non-stimulated and stimulated GI strips of rats and mice (*Mus musculus*) *in vitro* (9, 152) and gastric emptying *in vivo* (153).

Recent genome-wide analysis revealed that these mice and rats are species lacking genes for motilin and its receptor (10, 12). However, functional studies of recording GI motility indicated that MMC-like motility occurred at 15 min intervals in the stomach of fasting rats and mice, and that it was initiated by ghrelin and inhibited by a ghrelin receptor antagonist, suggesting that ghrelin, a family of motilin mediated the MMC-like motility in the rodents (154–156).

In the guinea-pig, however, the possible presence of motilin mRNA has been reported (52), and other studies indicated that motilin caused contraction of dispersed GI smooth muscle cells (157, 158), but isolated GI smooth muscle strips were insensitive to motilin (9, 11, 159). The discrepancy between the results in muscle strips and isolated cells might be explained as follows: motilin simulates both excitatory and inhibitory pathways in GI strips, and these opposite responses are cancelled and result in no responses (157). However, the recent re-examination demonstrated that motilin mRNA was not present and that the motilin deduced from mRNA (52) did not cause contraction and did not modify the neural responses in the guinea-pig GI tract (11). We also found that an MLN-R-like structure in the guinea-pig gene database but its homology with human MLN-R was very low (42.5%), suggesting that functional MLN-R might not exist in the guinea-pig (11). In the guinea-pig, if the motilin gene could be expressed and motilin is present in the duodenal mucosa, the MLN-R gene would be degenerated as in other rodents (10). Therefore, motilin may not have a GI regulatory function in the guinea-pigs.

Recording myoelectric activity in conscious guinea-pigs has indicated that the MMC-like myoelectric activity was elicited in the duodenum but not in the stomach, and it propagated toward the jejunum and ileum. These MMCs were not disrupted by feeding, but the frequency of the complex activity decreased by feeding (160). The characteristics of the myoelectric complex and the effects of motilin and ghrelin have not been examined.

### Humans

Similar to the GI motility pattern in dogs, GI motility in humans can be divided into distinct interdigestive and digestive contractions. Most of the spontaneous active front of the MMC in the interdigestive state originates in the stomach (16, 20, 161). Human motilin and the receptor have been identified (**Figure 1** and **Table 1**). As in dogs, motilin is thought to be the initiator of phase III of the gastric MMC because exogenous motilin causes MMC and because the plasma motilin concentration fluctuates in a cyclic manner in association with phase III of the MMC originating from the antrum (16, 161, 162). Janssens et al. (20) found that the active fronts of the MMC originating in the stomach were preceded by a motilin peak and that pancreatic polypeptide decreased the motilin levels and active fronts of the gastric MMC without affecting those of the intestinal MMC. Ondansetron, a 5-HT_3_ receptor antagonist, also decreased the cyclic increase of motilin and gastric phase III of MMC in the stomach, but it did not affect the MMC in the small intestine (163). Different inhibitory actions of atropine on the motilin-induced phase III activities in the antral and duodenum regions also suggest the different mechanisms of motilin-induced MMC in the stomach and small intestine: phase III activity of gastric MMC is dependent on muscarinic cholinergic mediation and the 5-HT_3_ receptors located on the vagus afferent neurons but that the contractile action of motilin in the duodenum involves a non-cholinergic mechanism (164). In addition, vagotomy abolished the MMC pattern in the stomach but had a minimal effect on the small intestinal MMC pattern (165). Therefore, the underlying mechanisms of the gastric MMC and intestinal MMC in humans are different, and motilin initiates only the phase III of the gastric MMC through activation of the 5-HT_3_ receptors and linked vago-vagal reflex pathway connecting enteric cholinergic neurons (**Figure 3**). Unlike in dogs, ghrelin causes an active front of phase III of the gastric MMC without changing the plasma motilin concentration in humans. However, the plasma ghrelin does not fluctuate like motilin in accordance with the gastric MMC and the role of ghrelin in regulation of the MMC has not been determined (111, 112). Recently, it was proposed that the MMC signals hunger sensation from the periphery to the brain in humans (111, 166). Therefore, motilin is a hunger hormone transporting a hunger signal through activation of vagus afferent neurons which also stimulate the vagus efferent neurons causing gastric phase III.

The *in vivo* GI motility-stimulating actions of motilin are similar in humans and dogs, but motilin stimulates contractility of human GI tract *in vitro*, in contrast to the isolated canine GI tract. 13-Nle-motilin caused contraction of the stomach and small intestine but not large intestine of humans, and atropine did not decrease the responses (9). Ludtke et al. (167) reported that the circular muscle strips are more sensitive to motilin than are longitudinal muscle strips in various regions of the stomach (pylorus, corpus, fundus, and antrum), and these contractions were resistant to tetrodotoxin and atropine, but duodenal strips were insensitive to motilin. These pharmacological studies indicated the presence of MLN-Rs on smooth muscle cells in a region-dependent manner. However, the results of a [^125^I]-labeled-motilin binding study in the human stomach showed the presence of MLN-Rs in both neural synaptosomes and smooth muscle membranes, and the binding in neural synaptosomes was dominant (58). As in the rabbit GI tract, different dissociation constants of MLN-R agonists suggest the presence of receptor subtypes located on smooth muscle and enteric neurons (58). However, the presence of MLN-R subtypes has not been clear at present. Such neural MLN-Rs have been also demonstrated by an immunohistochemical study, and 50-60% of cholinergic neurons were shown to have MLN-R immunoreactivities (168). A functional study using electrical field stimulation (EFS) showed enhancement of EFS-induced cholinergic contraction and increase in smooth muscle tonus by motilin or MLN-R agonists in the antrum with low activity in the fundus and small intestine. A high concentration of motilin is needed to increase smooth muscle tonus through activation of muscle MLN-Rs (168). Therefore, the results of the *in vitro* study clearly indicate the physiological importance of neural MLN-Rs on gastric cholinergic neurons as suggested by the results of the *in vivo* study (164). The neural MLN-Rs on gastric cholinergic neurons and the 5-HT_3_ receptors on afferent terminals of the vagus nerves are responsible for inducing atropine-sensitive phase III contraction of the MMC in the human stomach *in vivo*, whereas the role of myogenic MLN-Rs is not crucial because of their low affinity and/or low expression level compared to those of neural MLN-Rs (**Figure 3**).

### Rhesus Monkey

Rhesus monkeys (*Macaca mulatta*) have been used in *in vivo* and *in vitro* GI contraction studies to examine the effects of motilin-induced responses in comparison with those in humans.

When GI motility was recorded using force transducers, both interdigestive and digestive contraction patterns were observed (169). As in humans and dogs, interdigestive MMCs were observed in both the gastric antrum and duodenum at intervals of 120-150 min, and exogenous motilin caused the phase III-like actions of the gastric MMC, and which was decreased by hexamethonium but not by atropine. Therefore, motilin activates the neural pathway consisting of intrinsic cholinergic nerves, but ACh/muscarinic receptor is not a final mediator of phase III of the MMC, being different from human and canine gastric MMCs. An increase in gastric emptying by motilin was thought to be due to the gastric motility-stimulating action of motilin (169, 170). An *in vitro* study indicated that motilin preferentially caused contraction of the upper GI tract depending on the region-dependent distribution of MLN-Rs (169). Motilin function in rhesus monkeys is thought to be similar to those in humans, and rhesus monkey would be a useful animal model for investigating the physiological functions of motilin in humans.

### House Musk Shrew

In earlier motilin research, dogs (*in vivo*) and rabbits (*in vitro*) have been mainly used. However, these animals are hard to use for laboratory experiments because of their body sizes and different responses to motilin from those in humans. From these points of view, the house musk shrew (*Suncus*) is very useful. *Suncus* belongs to the order of insectivore, and its body size is similar to that of rats, making it easy to handle in experiments. Interestingly, *Suncus* has been used for the development of anti-emetic drugs because it can vomit differently from the rodents (171). Sanger et al. (12) reported that the motilin system is correlated with the ability to vomit with some species exceptions. *Suncus* motilin and ghrelin (44, 172) and their receptors (78) have been identified, and functions of motilin in regulation of GI motility have been investigated in both *in vivo* and *in vitro* (15, 71, 99, 173).

Motilin caused contraction of *Suncus* gastric strips in an *in vitro* study, and the contraction was abolished by atropine and tetrodotoxin and was significantly decreased by hexamethonium, phentolamine, ondansetron and naloxone. These results indicate that the motilin-induced contraction *in vitro* is mediated by a pure enteric neural pathway including cholinergic (nicotinic and muscarinic receptors), adrenergic (α-adrenergic receptor), serotonergic (5-HT_3_ receptor) and opiatenergic neurons (opiate receptor) (71).

The actions of motilin on gastric motility were also observed in an *in vivo* study using conscious free-moving *Suncus*. As in dogs and humans, the GI motility patterns could be divided into interdigestive and digestive patterns. During the interdigestive periods, the stomach and duodenum showed MMCs consisting of three different phases at intervals of 80-150 min, and the gastric MMCs propagated to the duodenum. Motilin and erythromycin caused phase III activity of the gastric MMC (15). The appearance of phase III activity was inhibited by an MLN-R antagonist, MA2029 (71).

The contribution of ghrelin to the regulation of the gastric MMC with motilin has been reported (173). Ghrelin enhances phase II activity of the MMC in a vagus nerve-dependent manner, and the duration and amplitude of phase II are attenuated by vagotomy. Motilin initiated phase III-like activity in the stomach in a vagus nerve-independent manner, and a ghrelin receptor antagonist or an MLN-R antagonist decreased the phase III activity of the gastric MMC. These results indicate that motilin is involved in the induction of phase III of gastric MMC as in humans, dogs and that ghrelin is involved in initiation of phase II and subsequently enhances motilin-mediated phase III contractions (173). Motilin mainly activates the enteric nervous system independently of its actions on vagus afferent neurons and smooth muscles, while ghrelin indirectly regulates phase III activity through its actions on vagus afferent neurons. Enhancement of phase III activity by ghrelin indicates a synergistic interaction of motilin and ghrelin in contraction of the *Suncus* stomach. Ghrelin decreased the GABAergic nerve-mediated inhibition in the myenteric plexus that caused enhancement of motilin-induced gastric phase III contraction (174).

A functional role of the vagus nerves in regulation of the motilin-induced response and synergistic action of ghrelin have also been demonstrated in a digestive state. In the vagotomized *Suncus*, postprandial irregular contractions were not observed, indicating the involvement of vagus nerves in the digestive contractions. In vagus nerve-intact animals, motilin does not cause contraction in the digestive state but causes contraction in vagotomized animals, indicating that the vagus nerves play a suppressive role to the action of motilin (173). However, the mechanisms have not been clarified yet.

The complicated regulation mechanisms of the gastric MMC by motilin and ghrelin were indicated for the first time by using *Suncus.* Measurements of plasma motilin and ghrelin concentrations during the gastric MMC might provide more information about the roles of motilin and involvement of both peptides in GI motility regulation.

### Opossum

The opossum (*Didelphis virginiana*) is a small animal with a body size similar to that of domestic cats, and it belongs to the order of Didelphidae. As shown in **Figure 1** and **Table 1**, opossum motilin and MLN-R have been identified.

GI electric activity has been measured in conscious opossums and was found to be different in the interdigestive and digestive periods. In fasted periods, cyclic myoelectric activity complexes migrating toward the jejunum were observed in the gastric antrum at 90-min intervals, and it was consisted of three phases as dogs and humans. They were disrupted by feeding and changed into irregular small continuous electrical activity (digestive contraction) (175).

The involvement of motilin in the regulation of migrating myoelectric activity in the opossum was examined. Plasma motilin concentration changed in a cyclic manner and the duration between two peaks was about 90 min, and the peak corresponded to phase III of myoelectric activity in the duodenum (104). Infusion of motilin (0.3-0.9 µg/kg/h) initiated phase III activity in the stomach and duodenum, and the activity propagated toward the jejunum like spontaneous phase III. Therefore, motilin is proposed to be a mediator of the phase III of MMC in the stomach or duodenum in the opossum (104).

### Pigs

Motilin was firstly identified in pigs (*Sus scrofa domesticus*) and motilin-immunopositive cells were localized in endocrine cells of the small intestine (1–3, 176).

It was reported that MMC was observed in the duodenum, not in the stomach, unlike those in dogs and humans (102, 177–180). However, the myoelectric complexes were not completely disrupted by feeding (179). An association between plasma motilin concentration and MMCs was not observed, and plasma motilin concentration was almost stable during MMCs (177). In addition, motilin infusion did not induce phase III-like activity and affected the interval of phase III activity (178). Infusion of acid into the duodenum increased motilin release, but the increased motilin did not produce the phase III-like activity (102). Immunoneutralization of motilin had no effects on appearance of the MMCs (181). Thus, in pigs, motilin is thought not to be a mediator of the MMCs.

An *in vitro* study indicated that motilin did not cause contraction of muscle strips and did not modify neural responses in the stomach and intestine (182). Little is actually known about the physiological roles of motilin in porcine GI function, although motilin was first discovered in pigs.

### Ruminants

MMCs have been reported in gastric antrum-duodenal regions of conscious sheep, and the interval between phase III of the myoelectric complex is approximately 120 min (183). Unlike in dogs and humans, the myoelectric activity is not changed by feeding (184). Plasma motilin concentration does not fluctuate and stays at almost the same level during an appearance of phase III (185). Infusion of motilin and its receptor agonist, erythromycin did not cause any changes in myoelectrical activity of antrum-duodenal regions, although a bolus application of them increased the myoelectric activity (183). These findings suggest that motilin is not a mediator of migrating myoelectric activity in sheep.

### Summary of Motilin Action in Mammals

The presence of the motilin system and characteristics of MMC/migrating myoelectrical activity in the stomach and small intestine, the effects of motilin on GI contractility in *in vivo* and *in vitro* experiments, and changes in the plasma motilin concentration during the MMC were summarized in **Table 3**.

**Table 3:** Summary of effects of motilin on gastrointestinal contraction in mammals and birds.

	Presence or absence of motilin system	Migrating motor (myoelectric) complex in the fasting period [stomach]	Migrating motor (myoelectric) complex in fasting period [small intestine]	Disruption of MMC by feeding	Action of motilin on GI motility		Plasma motilin concentration during MMC or ROCs
					*In vivo* study	*In vitro* study	
Human	Presence	Observed	Observed	Yes	Induction of gastric MMC. Increase in gastric emptying	Contraction	Cyclic change consistent with MMC
						Neural and myogenic	
Monkey	Presence	Observed	Observed	Yes	Induction of gastric MMC. Increase in gastric emptying	Myogenic contraction	Not available
Dog	Presence	Observed	Observed	Yes	Induction of gastric MMC. Increase in gastric emptying	Ineffective	Cyclic change consistent with MMC
Suncus	Presence	Observed	Observed	Yes	Induction of gastric MMC	Neural contraction	Not available
Rabbit	Presence	Not observed	Observed	No	No effect on jejunum MMC	Contraction	Not available
						Neural and myogenic
Opossum	Presence	Observed	Observed	Yes	Inducton of gastric MMC.	Not available	Cyclic change consistent with MMC
Guinea-pig	Absence	Not observed	Observed	No	Not determined	Ineffective	Motilin not present
Rat	Absence	Observed	Observed	Yes	Gastric MMC mediated by ghrelin	Ineffective	Motilin not present
Mouse	Absence	Observed	Observed	Yes	Gastric MMC mediated by ghrelin	Ineffective	Motilin not present
Pig	Presence	Not observed	Observed	No	No effect on duodenal MMC	Ineffective	No change during MMC
Sheep	Presence	Not observed	Observed	No	No effect on duodenal MMC	Not available	No change during MMC
Chicken	Presence	Not observed	MMC and rhythmic oscillating complex (ROCs) (fasting)	No	No effect on duodenal MMC. ROC is produced.	Contraction	High level during ROCs
						Neural and myogenic	

Motilin is thought to be a physiological mediator of the phase III of gastric MMC in humans, dogs, monkeys, *Suncus* and opossums, since they eat large meals with a low frequency, and they have clear fasting and digestive periods. In mammals with different feeding behaviors (small meals with a high frequency) such as rabbits, pigs and sheep, physiological roles of motilin in regulation of the GI motility have not been clearly understood. It is possible that motilin affects GI motility in the digestive state because MLN-R agonists, such as ABT-229, EM574 and GM116 increase the gastric emptying in humans, dogs and monkeys (170, 186–188). However, plasma motilin concentration is thought to be low in the digestive state and functional roles of endogenous motilin have not been examined. Motilin transmits a hunger signal from the periphery to brain in humans (166), and there might be a relationship among eating style, hunger signals and functions of motilin in the GI tract of mammals.

## GI Motility-Stimulating Action in Non-Mammals

### Birds

Isolated GI strips of chickens, quails and pheasants were used in *in vitro* contraction studies for motilin (33, 34, 82, 83, 189). Chicken or human motilin caused contraction of the small intestine (duodenum, jejunum and ileum) in the three avian species by activation of MLN-Rs on smooth muscles because tetrodotoxin or atropine failed to decrease the contraction. Rabbit duodenum and chicken intestine showed different contractile activities by human motilin and chicken motilin (**Figure 2**), and an MLN-R agonist, erythromycin did not cause contraction of avian intestine and an MLN-R antagonist, GM109 also failed to decrease the response of motilin in the chickens and pheasants, which is strongly suggestive of structural differences in avian MLN-Rs from mammalian MLN-Rs (33, 34, 82, 83). In fact, the chicken MLN-R has a quite different structure from those of human and rabbit MLN-Rs (81).

In chickens, quails and pheasants, motilin causes the strongest contraction in the small intestine followed by the proventriculus, but does not in the crop, gizzard, and colon (34, 82, 83, 189). This pattern of different ranking of responsiveness is common in three avian species. Contraction in the proventriculus was decreased by tetrodotoxin or atropine, being different from the response in the small intestine, suggesting that motilin acts on MLN-Rs located on enteric cholinergic nerves, which is consistent with the results in humans and rabbits (58, 145). These region-related different contraction mechanisms (ileum *vs.* proventriculus) are also common in the three avian species (34, 82, 83).

In *in vivo* studies, MMC is observed in the chicken GI tract (190–192) as in mammals. The chicken MMC is consisted of three phases, basic pattern of quiescence (phase I) and irregular spike activity (phase II) followed by intense regular spike activity (phase III). The frequency and duration of chicken MMC are similar with those in mammals, but the migrating velocity is slow. In addition, the avian migrating myoelectric activity originates from the duodenum, not the stomach, and it is not disrupted in the digestive states (190–193). The detail regulation of the MMCs in chickens has not been examined, but it is known that the appearance of myoelectric complex is modulated by some gut hormones including cholecystokinin and gastrin (191, 192). Rodriguez-Sinovas et al. (193) reported that motilin was not a mediator of phase III activity of MMCs in chicken because motilin did not induce phase III activity.

Rather than MMCs, a new pattern of electric activity called rhythmic oscillating complexes (ROCs) has been reported in the chicken small intestine (191, 194). ROCs are highly organized myoelectric events consisting of several intestinal spike bursts migrating downward (from the duodenum to ileocecorectal junction), followed by groups of upward spike bursts from the end of the small intestine to the gastric pylorus to mix intestinal luminal contents. It appears only in a fasted condition regardless of the phase of the myoelectric complex, and they drive the intestinal contents to the upper part of the GI tract including the stomach and duodenum (191, 194). ROCs have not been reported in mammals, but ROC-like contractions and retrograde giant contractions have been observed in mammals before vomiting (195). Rodriguez-Sinovas et al. (193) reported that plasma motilin concentration was high during spontaneous ROCs occurred in the chicken small intestine, and that exogenous motilin triggered the ROCs activities. This was the first indication of the involvement of motilin in the regulation of small intestinal ROCs in birds in the fasting periods.

In *in vitro* experiments, the responsiveness to motilin was high in the small intestine including the jejunum and ileum in all avian species examined (34, 82, 83), and the expression level of the MLN-R mRNAs was high in the ileum of adult chickens (196). These observations suggest that the small intestine is the major target of motilin in birds, and that motilin regulates the small intestinal contractility in a fasting state.

### Reptiles

Although motility of isolated GI strips of reptiles (*Burmese python*) has been measured (197), the effects of motilin on reptile GI contractility have not been examined yet despite the molecular evidence for the presence of motilin and MLN-Rs (**Figure 1** and **Table 1**). In our study, turtle and alligator motilins caused contraction of the rabbit duodenum and chicken ileum with low affinity compared with human motilin or chicken motilin (**Figures 2A, B**), indicating that reptile motilins can be agonists for mammalian and avian MLN-Rs. However, contraction studies using the GI tract of some reptiles themselves are necessary to determine that motilin is a regulator of GI contractility in reptiles.

### Amphibians

Our recent database searches have indicated the presence of a motilin-like peptide in newts and axolotl but not in frogs (**Table 1**), even though MLN-R is thought to be present both in newts and frogs (**Figure 1**).

In *in vitro* studies using isolated GI tract of frogs, human motilin caused contraction of stomach of the black-spotted pond frog (*Pelophylax nigromaculatus*) and the upper small intestine of the bullfrog (*Lithobates catesbeiana*) and tropical clawed frog (*Xenopus tropicalis*). However, other GI regions including the middle and lower intestines were insensitive (84, 85). Therefore, motilin sensitivity in frogs seems to be dependent on the GI region, as has been seen in other animals, and the motilin action in the frogs suggests the possible presence of MLN-R-like receptor. However, erythromycin or GM109 did not cause contraction or inhibition of motilin responses in the frog GI tract (85), suggesting that the structure of MLN-R-like receptor is different from that of mammals. In a database, an MLN-R candidate was found in the tropical clawed frog (XM 002935747), and homology of the Xenopus MLN-R with human MLN-R was relatively low (50%). Phylogenetic tree analysis of MLN-R clearly showed the different clade of the Xenopus MLN-R from mammalian MLN-R (**Figure 1**). The presence of MLN-R-like receptor might be responsible for human motilin causing a contraction, but endogenous motilin has not found in the *Xenopus*, suggesting that only the motilin gene, but not the MLN-R gene may have been lost during evolution of anuran amphibians. In contrast to the results of functional studies in the frogs, human motilin was ineffective in the upper small intestine of the Japanese fire belly newt (84). However, our recent study using the isolated stomach of the fire belly newt indicated that newt motilin caused a contraction of the gastric strips with high affinity compared with other motilin peptides (**Figure 2C**). Furthermore, small intestinal preparations (upper, middle, and lower intestines) were insensitive to newt motilin. These results indicate the presence of the motilin system in the newt which regulates GI motility in a region-dependent manner as seen in birds and mammals.

### Teleost Fish

Molecular studies demonstrated the presence of motilin and its receptor in teleost fish including zebrafish (*Danio rerio*) (45, 198), ballan wrasse (*Labrus Bergylta*) (199), spotted sea bass (*Lateolabrax Maculatus*) (8) and other species (**Figure 1** and **Table 1**).

In the intestinal bulb and middle or distal intestinal preparations of the zebrafish GI tract, human motilin caused a contraction (198). On the other hand, our study showed that zebrafish motilin caused only a very small contraction even at high concentrations (over 1 µM), though this peptide activated the zebrafish MLN-R expressed in HEK293 cells at much lower concentrations (3-100 nM) (86). The small contraction by zebrafish motilin *in vitro* would be responsible for the low expression level of the MLN-Rs, and it is thought that the motilin system is not a key regulator of intestinal motility in zebrafish (86). Considerable expression of both motilin and MLN-R have been demonstrated in the stomach of the ballan wrasse (199) and the intestine of the spotted sea bass (8), but a GI contraction study for motilin has not been performed in those fish. In the spotted sea bass, starvation regulated the expression level of the motilin gene, and motilin enhanced the mRNA expression of ghrelin, gastrin, and cholecystokinin (8). These results suggest that motilin affects the expression of the other gut hormones related to digestion and energy homeostasis in fish instead of the regulation of GI motility.

### Summary of Motilin Actions in Non-Mammals

Both motilin and/or MLN-R are present in almost all non-mammalian vertebrates except anuran amphibians (frogs). Motilin is less effective in causing GI contraction in fish, but it appears to cause contraction from the amphibian and avian GI tracts in a region-related manner: the stomach and upper intestine are sensitive to motilin in amphibian, but the entire small intestine is highly responsive to motilin in avian species. Through studies in non-mammals, it can be seen for the first time that the GI motility-stimulating action of motilin is not common in vertebrates since motilin stimulates GI contraction in birds and amphibians but not in fish.

## Functions of Motilin in Peripheral Organs Other Than GI Tract and Brain

Although the number of studies has been limited, other biological actions in digestive function and in other organs including the blood vessels and brain have been reported (**Table 4**).

**Table 4:** Effects of motilin in mammals other than its gastrointestinal motlity-stimulating actions.

Target sites	Effects (animals)	References
Central nervous system	Increase in food intake (mouse, rat)	(200, 201)
	Anxiolytic behavior (mouse)	(202)
	Increase in growth hormone release (rat)	(203)
	Decrease in urinary bladder contraction (rat, icv)	(204)
	Depolarization of Purkinje cells (rat)	(205)
	Increase in c-fos expression of supraoptic nuclei and paraventricular nuclei (rat)	(206)
	Increase in neural activity of the amyglada (rat)	(207)
	Decrease in neural activity of the lateral vesitbular nucleus (rabbit)	(208)
Cardiovascular system	Relaxation of blood vessels (dog)	(209)
	Relaxation of aortic valve (pig)	(210)
	Vasodilation of gastric blood flow (dog)	(211)
	Vasodilation (rat)	(212)
	No effects on heart rate (dog)	(211)
	No effects on heart rate (rat)	(212)
Endocrine/Exocrine system	Increase in gastric acid release (dog and suncus)	(213, 214)
	Increase in pepsinogen release (suncus)	(215)
	Increase in insulin release (dog)	(216, 217)
	Increase in pancreatic water, bicarbonate and protein release (dog)	(218)
	Decrease in ghrelin release (dog)	(14)
	Increase in somatostatin release (dog)	(219)
Intestinal mucosa	Increase in L-leucine absorption (rabbit)	(151)
	Decrease in L-proline absorption (rat)	(220)
Gallbladder	Contraction (dog, human, opposum)	(104, 221, 222)
Oesophagus	Contraction of lower esophageal sphincter (dog)	(223)

Motilin regulates the exocrine and endocrine functions, and stimulates the release of gastric acid, pepsinogen, insulin, somatostatin and pancreatic bicarbonate/protein (213–215, 218, 219). Motilin controls the cyclic release of insulin in fasted dogs. A comparison of the action of motilin in isolated islet β-cells and in conscious dogs suggests that motilin stimulates 5-HT release, and 5-HT activates the vago-vagal reflex through activation of the 5-HT_3_ receptors on vagal afferent terminals, and the vagal efferent stimulates ACh release, and which activates the muscarinic receptors on islet β-cells (216, 217). On the other hand, insulin that is released by glucose after feeding decreases motilin release (93), suggesting the presence of glucose- and insulin-related negative feedback for motilin release. In addition, motilin decreases the release of ghrelin in the dog stomach (14).

In the cardiovascular system, motilin shows increase in blood flow in dogs (209, 211). MLN-R is dominantly expressed on the endothelium of gastric artery and the motilin-induced increase in blood flow is selective for gastric artery. Therefore motilin regulates both gastric blood flow and motility simultaneously (211). The endothelial cells-dependent relaxation by motilin was also reported in the porcine aortic valvular strips (210).

Motilin is thought to act in the CNS because motilin-immunoreactive cells were present in the brains of dogs, pigs and monkeys (26, 42, 224, 225), and because MLN-R was also detected in the brains of humans and rabbits (23, 24, 118). But there are only a few functional studies: Chan-Palay et al. (208) reported a decrease in neural activity of the lateral vestibular nucleus by motilin in rabbits; the central actions of motilin have been discussed in zebrafish because of high expression of MLN-R mRNA in the brain (45).

Rats and mice lack motilin system but central and peripheral actions of motilin have been reported (**Table 4**). Motilin stimulates the growth hormone release (203) and feeding (200, 201). Chen et al. (205) reported that motilin caused depolarization of rat cerebellum Purkinje cells. Increased neural activity in the amygdala (207) and c-fos expression of supraoptic nuclei and paraventricular nuclei in the hypothalamus have been reported (206). Motilin applied to the CNS decreased bladder contraction (204) and increased gastric motility in rats (207). In peripheral organs, motilin caused the vasodilation without changing heart rate in rats (212) and inhibited proline absorption in the rat jejunum (220). These motilin responses in rats and mice could be actions on a non-MLN-R that recognizes the sequence of motilin, but the non-MLN-R and its endogenous ligand have not been identified.

## Conclusion

This review summarized the distribution, structure, receptor expression and function of motilin, with a focus on the GI motility-stimulatory action of motilin in a range of species including fish to mammals.

Motilin and MLN-R are present in almost all vertebrates, and their structures have diversified during evolution. A highly conserved N-terminal commencing the amino acid indicated by phenylalanine is thought to be essential for biological activity in mammalian/avian motilin lineage. Reptile motilin is considered to be in the transition stage to mammalian/avian type, whereas the sequences of fish and amphibian motilins differ significantly. In the molecular evolution of motilin, there may have been a major event at the time the reptiles emerged. The differences in motilin sequences are due to mutations in protein coding domains during species evolution which were probably motivated by adaption. The C-terminal sequence is more conserved than that of the N-terminal, suggesting that the C-terminal may exert an as yet unknown function in addition to stimulation of GI motility as mediated *via* the N-terminal.

GI motility stimulation in a region-specific manner is the main action of motilin, and motilin is the predominant mediator of the phase III interdigestive MMC at least in humans, dogs, monkeys, opossum and Suncus. MLN-Rs mediating GI contraction located on both smooth muscle cells and on enteric neurons, and 5-HT released by motilin activates the vago-vagal reflex pathways. Contribution of these pathways diversified from species to species, even in mammals, and it is thought to reflect the evolution of animals and their feeding behavior. Motilin doesn’t seem to regulate GI motility in fish, but has acquired a GI motility regulatory function in urodele amphibians, and that function would have been passed down to birds and mammals.

It is interesting to anticipate the changes of motilin actions with consideration of vertebrate evolution. There are three questions. One is what is the preliminary action of motilin in fish if it is not GI motility? The distribution of the receptors may hold the answer to this question. Secondly, if the primary function was not related to GI motility, why did it come to regulate GI motility? Finally, why is expression of the motilin gene lost in anuran amphibians whereas expression of the receptor remains?　This brings the question as to whether this receptor retains some biological function *in vivo*. By cross-species comparisons, it is envisaged that further understanding and answers to these queries may be addressed.

## Author Contributions

All authors contributed to the article and approved the submitted version.

## Funding

This study was partly supported by JSPS-Japan KAKENHI Grant number 23570081 and 26440169 to TK and Grant number 26440174 to HK and by Grants-in-Aid to Cooperative Research from Rakuno Gakuen University 2014 (2014–14).

## Conflict of Interest

The authors declare that the research was conducted in the absence of any commercial or financial relationships that could be construed as a potential conflict of interest.

## Publisher’s Note

All claims expressed in this article are solely those of the authors and do not necessarily represent those of their affiliated organizations, or those of the publisher, the editors and the reviewers. Any product that may be evaluated in this article, or claim that may be made by its manufacturer, is not guaranteed or endorsed by the publisher.
